# Specific Streptomyces strain enhances the growth, defensive mechanism, and fruit quality of cucumber by minimizing its fertilizer consumption

**DOI:** 10.1186/s12870-023-04259-y

**Published:** 2023-05-11

**Authors:** Elham Orouji, Mohammad Fathi Ghare baba, Akram Sadeghi, Shahrokh Gharanjik, Parisa Koobaz

**Affiliations:** 1 Department of Plant Breeding and Biotechnology, Faculty of Agricultural Engineering, Shahrood University of Technology, Shahrood, Iran; 2 Department of Molecular Physiology, Agricultural Biotechnology Research Institute of Iran (ABRII), Agricultural Research, Education and Extension Organization (AREEO), Karaj, Iran; 3 Department of Microbial Biotechnology, Agricultural Biotechnology Research Institute of Iran (ABRII), Agricultural Research, Education and Extension Organization (AREEO), Karaj, Iran

**Keywords:** Plant growth promoting rhizobacteria, Resistance, *Streptomyces*

## Abstract

**Background:**

The required amounts of chemical fertilizers (NPK) are determined by plant yield, and product quality is given less consideration. The use of PGPRs is an environmentally friendly approach that, in addition to increasing yield, also improves fruit quality. This study examined the role of specific *Streptomyces* strains in aiding cucumber plants to 1) use fewer NPK fertilizers in the same quantity 2) improve the quality of cucumber fruit, and 3) promote growth and defense system.

**Results:**

In this study, the effect of 17 *Streptomyces* strains on the vegetative traits of cucumber seedlings of the Sultan cultivar was evaluated as the first test. Four strains of *Streptomyces* with the highest root and shoot dry weight were selected from the strains. This experiment was performed to determine the interaction effect of selected strains and different amounts of NPK on cucumber yield, quality, physiological and biochemical responses of plants. The first experiment’s results revealed that strains IC6, Y7, SS12, and SS14 increased significantly in all traits compared to the control, while the other strains dramatically improved several characteristics. Analysis of variance (ANOVA) revealed significant differences between the effect of strains, NPK concentrations, and their interactions on plant traits. The treatments containing 75% NPK + SS12, yielded the most fruit (40% more than the inoculated control). Antioxidant enzymes assay showed that SS12 substantially increased the activity of POX, PPO, and the expression of the genes related to these two enzymes. Hormone assay utilizing HPLC analysis revealed that various strains employ a specific mechanism to improve the immune system of plants.

**Conclusions:**

Treatment with strain SS12 led to the production of cucumbers with the highest quality by reducing the amount of nitrate, and soluble sugars and increasing the amount of antioxidants and firmness compared to other treatments. A specific *Streptomyces* strain could reduce 25% of NPK fertilizer during the vegetative and reproductive growth period. Moreover, this strain protected plants against possible pathogens and adverse environmental factors through the ISR and SAR systems.

## Introduction

The cucumber (*Cucumis sativus*) is one of the most widely consumed vegetables in the world. Based on annual reports from the Iranian Ministry of Agriculture-Jihad (Anonymous. Annual Agricultural Statistics. Ministry of Jihad-e- agriculture of Iran; 2020), the area under cultivation of cucumber greenhouses has increased during recent years. According to the Food and Agriculture Organization (FAO), cucumber production increased significantly from 0.66 million tons in 2019 to 1.2 million tons in 2020 (FAO, 2020, http://www.fao.org/faostat/en/#data/QC).

Each year, Iranian farmers add more than 5 tons per hectare of organic fertilizer and NPK fertilizers with varying ratios to cucumber greenhouse soil before cultivation. The cost of fertilizer use in commercial greenhouses in Iran accounts for about 20% of the total greenhouse income [1]. The excessive use of organic and chemical nitrate sources to achieve maximum yield in the greenhouse results in the high-level accumulation of nitrate in vegetables [2].

Nitrate can be reduced in the human digestve system and converted to nitrite, a toxic substance. Therefore, there are restrictions on consuming nitrate-containing foods [3]. Phosphorus overdose causes Zinc antagonism which reduces yield and fruit quality [4]. Additionally, potassium overdose may cause depression [5]. Using plant growth-promoting *Streptomyces* (PGPS) strains could reduce fertilizer consumption without reducing yield [6].

Iranian people prefer thin-skinned Persian cucumbers, which are between 10–15 cm long, about 100 g in weight and remain quite narrow. These baby cucumbers are nearly seedless and crispier than watery. Therefore, as an indicator of yield, marketable size, firmness and the aforementioned qualitative characteristics are essential. Fertilizers are one of the agents used to produce more crops. Chemical fertilizers, in particular, can increase crop yields, but their excessive negative environmental effects, such as soil acidification and nutrient loss have been well-documented [7, 8]. Organic fertilizer is a widely utilized strategy to increase crop yield, mitigate climate change [9, 10] and improve soil fertility and sustainability [11, 12]. The use of plant growth promoting rhizobacteria (PGPR) could be a viable alternative. However, the rate of crop yield increase when only organic fertilizers are applied is low [13–15]. Alternatively, the combined use of chemical and organic fertilizers has been proposed to meet the requirements of sustaining crop yield adequately [16]. Different types of PGPR genera colonize the root systems of plants and stimulate plant growth via direct or indirect mechanisms. The direct method includes biofertilization, root growth stimulation, rhizo-remediation, and plant stress control, whereas the indirect method includes induction of systemic resistance, competition for nutrients, and biological control to reduce disease levels [17, 18]. *Streptomyces* is a Gram-positive bacteria with an exceptional ability to survive in extreme environments such as salt and drought stress [19–21]. These microorganisms exhibit plant growth promotion combined with biocontrol properties [22–25]. However, very little is known about the effects of PGPR strains applied alone or in combination with various fertilizer doses on cucumber yield [26, 27]. As a plant growth promoter on pepper plants, *Streptomyces* PRIO41 demonstrated the ability to improve vegetative characteristics and biocontrol agent *Fusarium spp.* under two leveles of fertilization [26]. It was reported that *Streptomyces* strains promote plant growth by producing indole-3-acetic acid (IAA), 1-aminocyclopropane-1-carboxylate (ACC) deaminase activity and release of phosphate [28]. Two *Streptomyces* strains, IC10 and Y28, were closely related to *Streptomyces enissocaesilis* and *Streptomyces rochei* exhibited diazotrophic nitrogen fixation, siderophore production, solubilization of mineral phosphates, and production of indole-3-acetic acid at a high rate. They increased shoot length and shoot fresh and dry weight of tomato seedlings compared to those not inoculated [29]. Several studies demonstrate the positive effects of *Streptomyces* as PGPR on tomato fruits but none on cucumbers [30]. Another role of PGPR strains is to enhance plant growth by inducing a defense system to deal with biotic and abiotic stresses that limit plant growth [31]. The induction of plant resistance mechanisms by PGPR increases the production of defense-related enzymes or genes and stress-related plant regulators in various crops, comparable to pathogen defense mechanisms [32]. Peroxidases (POXs) [33] and polyphenol oxidases (PPOs) are the enzymes that are involved in the production of phytoalexins and phenolic compounds [34]. These plant responses to microbes are regulated through induced resistance (IR). Induced resistance is an enhanced defensive physiological reaction elicited by specific environmental stimulation. The two most clearly defined forms of induced resistance are systemic acquired resistance (SAR) and induced systemic resistance (ISR). Several rhizobacteria trigger the salicylic acid (SA)-dependent SAR pathway by producing SA, whereas other rhizobacteria, such as nonpathogenic microorganisms, use ISR and are dependent on jasmonic acid (JA) and ethylene signaling (ET). A combination of ISR and SAR can extend protection to a broader spectrum of pathogens than ISR or SAR alone [35, 36]. Although the effect of *Streptomyces* on plant growth has been demonstrated, the effect of specific PGPR on vegetative and reproductive cucumber growth and their role in reducing NPK fertilizer remains unknown.

Growth-promoting *Streptomyces* increased yield efficiency, but their role in increasing the absorption of chemical fertilizers and stimulating the immune system was not well known. Even the functional difference between different strains was a topic that needed to be addressed. The overall objectives of the study were introduced as follows: 1) screen PGPRs isolated from vegetable greenhouse soils to induce vegetative growth. 2) Detection the best strains to reduce NPK fertilizer consumption. 3) Investigation the effect of superior strains on qualitative fruit characteristics 4) Evaluation of defense-related enzymes (PPO and POX) activity, hormones (IAA, ABA and salicylic acid) content and expression of their related genes upon treatment of plants with *Streptomyces* strains.

## Material and methods

### Bacteria and inoculation form on cucumber seedlings

*Streptomyces* strains were previously isolated from cucumber rhizosphere soil [29] and investigated for growth on nitrogen-free medium, phosphorus solubilization, auxin, siderophore production and antagonistic activity against *Phytophthora. capsici* (Pc) and *P. drechsleri* (Pd) [20, 29]. For the bacterial treatments, *Streptomyces* cells and spores were centrifuged for 15 min at 8000 rpm, and then the pellet was suspended in 10 mL of sterile saline solution. Autoclaved sand was added to the bacterial suspension, and the final colony- forming units per gram of sand (cfu/g) was adjusted to 10^8^. Cucumber seeds (*Cucumis sativus* L. Sultan cultivar) were germinated in petri dishes after being washed with tap water (to remove traces of fungicide) and kept at 25˚C under dark conditions for 48 h before being transferred to light. After a week, all of the uniform seedlings were placed into cell plug trays (9 × 5 × 5 cm deep) filled with sterilized soil and peat moss (1:2), containing 2 g of sand containing bacteria per kilogram (kg) of soil, with one seedling in each cell. Sterilized sand without bacteria was used as a negative control. Tap water was used to water the seedlings while they were maintained at 27˚C and 16 h of light/8 h of darkness.

**Table 1:** The list of primers and their length were used in this study

**Target gene**	**Sequence**	**Length (bp)**
*ACS2*	F: TTTCTCCTCCGACGAGTTCA	168
	R: CGAGCGGTGGTGACGACTT	
*NCED3*	F: GCTATGGCTCTTGATGCGGT	212
	R: GGTTCGTGAAGTGGGTTGG	
*POX*	F: CTTAGCTCCACCTTCTATGACA	229
	R: ATTGAAACCCGTGATGCCTCC	
*ACT*	F: TCCACGAGACTACCTACAACTC	122
	R: GCTCATACGGTCAGCGAT	
*PPO*	F: CCAAAGAAATCGAGAAGCA	165
	R: AAAACTGCCCGCAAATTC	

### Vegetative growth screening of inoculated seedlings

For the first test, plants were harvested 4 weeks after inoculation. Their roots were washed, and their root and shoot fresh weight (RFW & SFW) were weighted immediately after sampling. Their root and shoot dry weight (RDW & SDW) were determined after drying in an oven at 70˚C.

### Greenhouse experiments with selected strains and different NPK levels

Four selected strains, namely Y7, IC6, SS12 and SS14, with the most significant effect on vegetative growth were chosen for the second experiment. The second experiment’s inoculum and seedling preparations were done as in the first test. After 4 weeks, the plants were transferred to 3 kg pots containing sterilized soil and peat moss (1:2). Two grams of sand containing bacteria were added to each kg of soil as the initial step in evaluating the effect of PGPR on reducing NPK fertilizer consumption and proper yield. To assess the effects of bacterial strains on cucumber plants under different NPK fertilizers, the experiments were set up as a factorial in the framework of a randomized complete block (RCB) with two factors: bacteria treatments (Y7, IC6, SS12 and SS14 and control containing sand without PGPR strain) and levels of chemical fertilizers (NPK: NO_3_, P_2_O_5_, K_2_O), 100% (2 g/l), 75% (1.4 g/l), 50% (1 g/l NPK fertilizer) with three replications. Initially, NPK fertilizer was used at 20:20:20. Then, at the flowering level, 10:52:10 was used and NPK with the high K level (20:20:32) was used to obtain the actual yield. The use of fertilizers started 1 week after transferring seedlings to the pots. Cucumber plants were kept in a greenhouse at 27 ± 2˚C and 16 h light/8 h darkness. Calcium chloride and other mineral compounds were used equally in all treatments. Fruits were harvested six times (45 days after treatment for the first time up to 60 days after treatment), and the number of fruits, length, yield, and firmness were recorded immediately after harvest. Finally, the roots were washed and weighed. In addition, fresh fruit weight was recorded and frozen for carotenoid, anthocyanin and ascorbate assays. Fresh fruits were sliced and dried in an oven at 55˚C to evaluate the percentage of dry weight and were then powdered through a mixer. Fruit dry powder was used to measure carbohydrates, nitrate, phosphate, and potassium.

### Greenhouse experiments with selected strains to evaluate biochemical and molecular properties

For the third test, four selected strains with the most significant effect on vegetative growth, i.e., Y7, IC6, SS12, and SS14, were used for molecular and biochemical experiments. The third experiment followed a similar procedure to the first. The leaves of four samples were harvested 96 h after bacterial treatment, frozen in liquid nitrogen and kept at -80˚C for further analysis. These materials were evaluated for total protein, APX, PPO activity, IAA, ABA, salicylic acid level hormones, and their gene expression.

### Protein and antioxidant enzymes activity

Frozen leaf samples (100 mg fresh weight) with 10 mg polyvinylpyrrolidone (PVP) were homogenized in 1 mL Na-Pi buffer (1 mM, pH 7). The homogenate was centrifuged at 12,000 rpm for 15 min. All operations were performed at 4˚C. The supernatant was used for protein and crude enzyme extract. The protein content was measured using the Bradford method [37] and the protein absorbance was measured at 595 nm using a spectrophotometer (Carry 300, USA).

The supernatants collected from the leaves of the third experiment were used to measure peroxidase (POX: EC 1.11.1.7) and polyphenol oxidase (PPO: EC 1.10.3.1) activity. The extraction and assay of POX were performed using a method modified by Chance and Mehley [38]. The reaction mixture contained 0.25 mL of 0.1 M phosphate buffer (pH 7.0), 0.25 mL 10 mM guaiacol, 0.03 mL H_2_O_2_, and 0.020 mL supernatant and deionized water (up to 1 mL reaction mixture). Enzyme activity was detected by an increase in the absorbance at 470 nm in the spectrophotometer (Carry 300, United States).

The PPO activity was conducted using a modified version of the method proposed by Esterbauer et al. [39]. Enzyme activity was expressed based on changes in absorbance per mg of total protein.

### Hormone assay

Leaves of samples (4 g fresh weight) were used for abscisic acid and indole acetic acid detection via the method suggested by Kelen et al. [40]. The dry residue containing hormones was dissolved in 1 mL of acidic methanol, filtered through 0.4 μ filters, and 20 μL was injected into an HPLC (Agilent, USA) on a C18 column. Acetic acid 0.2% and absolute methanol (50/50 v/v) with a flow rate of 0.7 mL/min were used as the mobile phase. Abscisic acid and indole acetic acid were detected using a UV detector with a fixed wavelength at 230 and 280 nm, respectively based on their corresponding standards.

Salicylic acid was extracted from the leaf fresh weight of samples (2 ml for 1 g powder) using acidic methanol (1/1000 v/v). Samples were shaken under dark conditions at 20˚C overnight. The homogenates were centrifuged at 10,000 rpm for 15 min. The supernatant was filtered and injected to HPLC (Agilent, USA) using a C18 column. Acidic methanol (1/1000 v/v) with a flow rate of 0.7 mL/min was used as the mobile phase. Salicylic acid standards were prepared to detect and measure at 280 nm [41].

### Growth parameters, fruit quality and dry weight percentage

Growth parameters such as fresh and dry weight of root, shoot, and fruit, the number of marketable fruit, and yield, were measured. Then, 20 g of cucumber was dried in the oven below 50˚C to determine the dry matter percentage. Firmness texture was evaluated using a texture analyzer (Hounsfield Test Equipment, UK) with a 500 N load cell. All fruit samples per control and treatments were punctured with a 7 mm diameter ball probe at a speed of 12 mm/s at their geometric center, and the results were expressed as N/mm [42].

### Chlorophylls and carotenoid content, glucose, sucrose, fructose, nitrate, phosphate, and potassium assays in fruit

Chlorophylls and carotenoid contents were measured via the Wellburn method [43]. Soluble carbohydrates were extracted from 0.03 g of fruit powder and measured following the method of Dubois et al. [44] with some modifications. The supernatant was evaporated, and the pellet was re-dissolved in HPLC grade distilled water before application to a reversed phase HPLC (Knauer, Germany) on a Eurokat H column (300 × 80 mm, 10 μm particle size). Acidic water (containing sulfuric acid pH = 2.5) with a flow rate of 1 mL min^−1^ was used as mobile phase. RI detector detected glucose, sucrose, and fructose and the peak areas were compared to their respective standards to determine sugar contents. The total anthocyanin concentration was measured by weighing 0.1 g of fresh fruit using a modified version of the method developed by the method of Fuleki and Francis [45]. The absorbance of anthocyanin content was detected at 550 nm by using an extinction coefficient of 33,000 M^−1^ cm^−1^ (Carry 300, USA).

Total ascorbic acid (ascorbic acid + dehydroascorbic acid) was determined using the method of Al-Ani et al. [46]. Reduced ascorbate is obtained by reducing dehydroascorbic acid from total ascorbate.

Nitrate assay was conducted according to the method described by Hachiya and Okamoto [47] using 0.1 g of dried fruit powder. The calibration curve was drawn using salicylic acid, and the maximum absorption was read at 410 nm. The phosphate assay was performed based on Murphy and Riley’s *method* [48], using supernatant extraction from nitrate measurement. To measure potassium, 0.01 g of dried cucumber fruit was utilized. Soluble K was extracted using the BWB method (BWB9), and the determination was made using flame photometer according to the manufacturer’s instructions ( A guide to flame photometer analysis, United Kingdom, 2012) 

### Quantitative real-time PCR analysis of defense system and hormone related genes

Total RNA was extracted from leaves using TRIzol™ (Invitrogen protocol), and cDNA was synthesized using 1 mg of each RNA sample after treating with RNase-free DNase I (Fermentase, USA) using the Revert Aid First Strand cDNA Synthesis kit (Thermo, USA) based on the instructions provided. Quantitative PCR was performed in a 25 μL reaction containing 1 μL of template cDNA, 0.5 μL of 10 pM of each forward and reverse specific primer (Table 1), and the iQ SYBR Green Supermix kit (BioRad, USA) on a Roche multicolor real-time PCR detection system. The results were expressed as the normalized ratio of mRNA level of target gene to internal control Actin gene (ACT). The data were analyzed using the Relative Expression Software Tool (REST 2009) through the Pfaffi method [49].

### Statistical analysis

Statistical analysis of the first experiment was performed using analysis of variance (ANOVA) with ten biological replicates used for every treatment. The experimental design for the second experiment was a randomized complete block based on a factorial arrangement with three biological replications per treatment. The third experiment was analyzed using ANOVA with four biological replicates. All the data obtained were analyzed using SPSS version 22.0 (SPSS Inc. Chicago, IL, United States). Means were compared using Duncan’s multiple range test at the 1% probability level (*P*-value ≤ 0.01). Mean values were obtained from four biological replicates in the Real-time PCR method and the results were analyzed using the REST Software version 2009.

## Results

### Cucumber growth promotion activity of *Streptomyces* strains on cucumber seedlings growth

**Fig. 1:**
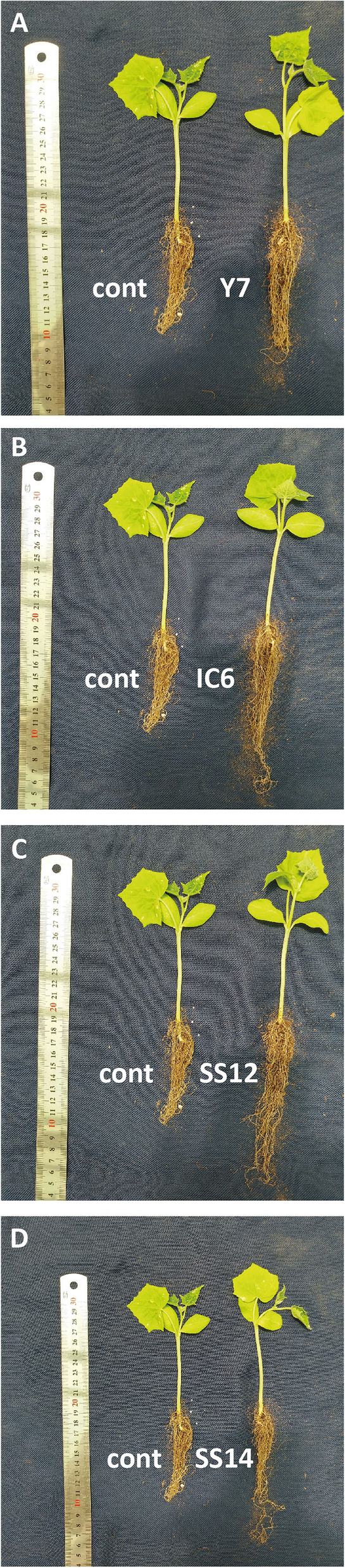
Vegetative characters of cucumber seedlings after 4 weeks inoculation compared to control. **A** Comparison of seedlings inoculated with Y7 strain. **B** Comparison of seedlings inoculated with IC6 strain. **C** Comparison of seedlings inoculated with SS12 strain. **D** Comparison of seedlings inoculated with SS14 strain

The results of analysis variance on the growth parameters of cucumber 7 day seedlings revealed that inoculation with six isolates significantly increased the fresh and dry weight of root plants 4 weeks after inoculation. Additionally, eight isolates were able to induce shoot growth of treated plants compared to the control. Among the 17 isolates tested, four strains (IC6, Y7, SS14, and SS12) exhibited a statistically significant difference in all measured characteristics, while the remaining isolates showed a marked increase in two or three measured characteristics compared to the control (Fig. 1). These four isolates increased root and shoot dry weight by approximately four and three times, respectively (Table 2). Subsequently, these four strains mentioned above were chosen for the next test.

**Table 2:** The effect of *Streptomyces* strains on vegetative characters of cucumber

Bacteria	Root		Shoot	
	Fresh weight (g)	Dry Weight (g)	Fresh weight (g)	Dry Weight (g)
K40	1.06 ± 0.07^ g^	0.22 ± 0.01^d^	10.4 ± 0.05^b^	1.44 ± 0.004^b^
IC10	1.34 ± 0.1 ^f^	0.20 ± 0.02^d^	4.99 ± 0.2^e^	0.43 ± 0.02^f^
IC14	1.27 ± 0.1^f^	0.23 ± 0.02^d^	5.36 ± 0.1^e^	0.46 ± 0.02^f^
IS6	1.24 ± 0.1^f^	0.19 ± 0.01^d^	5.00 ± 0.1^e^	0.41 ± 0.01^f^
IC19	1.48 ± 0.1^ef^	0.23 ± 0.01^d^	4.72 ± 0.2^f^	0.41 ± 0.03^f^
IC15	1.63 ± 0.1^e^	0.25 ± 0.02^d^	5.78 ± 0.2^e^	0.51 ± 0.04^e^
SSUP	1.62 ± 0.1^e^	0.22 ± 0.02^d^	4.84 ± 0.1^f^	0.36 ± 0.01^ fg^
Control	2.1 ± 0.1^d^	0.25 ± 0.02^d^	5.59 ± 0.3^e^	0.5 ± 0.03^e^
SS13	2.05 ± 0.1^d^	0.2 ± 0.06^d^	10.72 ± 0.2^a^	1.73 ± 0.02^a^
IS1	1.33 ± 0.1^ cd^	0.26 ± 0.05^d^	9.13 ± 0.2^c^	1.14 ± 0.02^c^
Y18	2.01 ± 0.1^d^	0.22 ± 0.04^d^	5.16 ± 0.2^e^	0.5 ± 0.02^e^
IS29	2.75 ± 0.2^b^	0.22 ± 0.06^bc^	5.62 ± 0.5^e^	0.5 ± 0.07^e^
IS17	2.93 ± 0.1^a^	0.25 ± 0.02^d^	5.4 ± 0.2^e^	0.5 ± 0.03^e^
SS14	2.51 ± 0.1^c^	1.00 ± 0.06^b^	10.35 ± 0.2^b^	1.36 ± 0.02^b^
IC6	2.7 ± 0.2^b^	0.98 ± 0.04^b^	10.06 ± 0.1^bc^	1.29 ± 0.03^bc^
Y7	2.78 ± 0.1^b^	0.95 ± 0.06^bc^	10.52 ± 0.2^b^	1.38 ± 0.03^b^
SS12	2.92 ± 0.05^a^	1.20 ± 0.01^a^	10.45 ± 0.05^b^	1.48 ± 0.004^b^

Values are the means (averaged from 10 replicates) ± SE. The same letters within each column represent non-significant differences according to Duncan’s Multiple Range Test (*P* < 0.05)

As shown in Table 3, these four strains had favorable PGPR properties including growth on nitrogen free medium, inorganic phosphorus solubilization, siderophore, and IAA production. Additionally, their antagonistic activity against *Phytophthora species* differed.

**Table 3:** PGP properties and antagonistic activity against plant phytopathogens

**Isolate**	**PGP activity**				**Antagonistic activity (%)**		**Accession No**
	Growth on N free Medium	Inorganic Phosphorus solubilization	Siderophore production	IAA production (μg/ml)	PD	PC	
IC6	+	+	+	9.2 ± 0.0	49	69	MH041276
Y7	+	+	-	7.7 ± 0.9	10	-	MH041461
SS12	+	+	+	24.1 ± 1.9	30	-	MG706140
SS14	+	-	+	24.5 ± 0.9	63	19	MH041316

*PC* Phytophthora capsici, *PD* Phytophthora drechsleri and genotypic characterization of 4 *Streptomyces* strains selected for greenhouse experiments (extracted from Abbasi et al., 2019 [29] and Abbasi et al., 2020 [20])

### Cucumber growth promotion activity of superior strains in a greenhouse under different fertilizer quantities

#### PGPR inoculation, fertilizer, and marketing characters

**Fig. 2:**
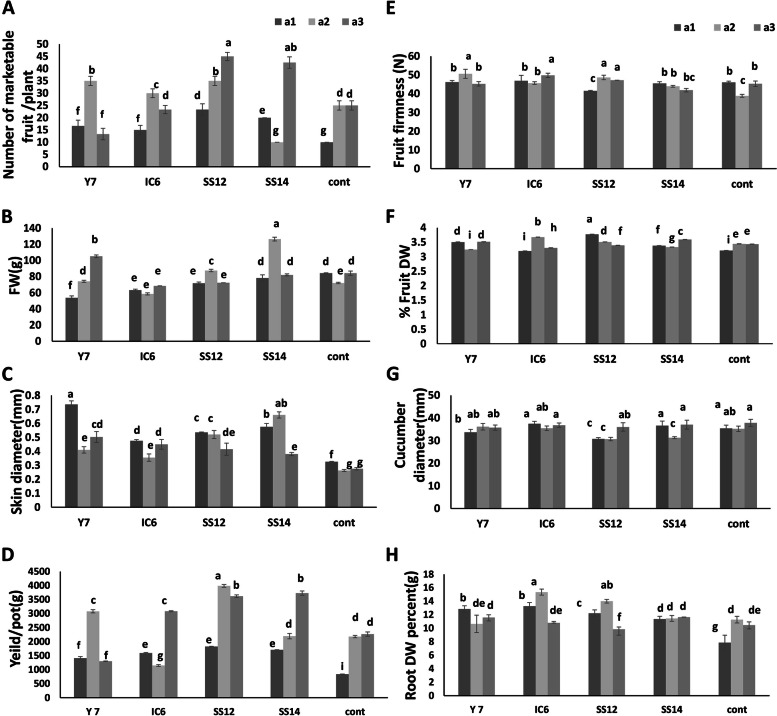
The effect of different quantities of NPK fertilizer and PGPR bacteria on **A** number of marketable fruits **B** fruit fresh weight **C** skin diameter **D** yield **E** firmness **F** fruit dry weight percent **G** cucumber diameter **H** root dry weight percent. Different levels of NPK fertilizers are a1 (50% = 1 g/L) a2 (75% = 1.5 g/L) and a3 (100% = 2 g/L) based on recommended amount of different growth levels of cucumber (vegetative growth, flowering, and fruiting). Values represent means of three replicates; bars represent standard deviation of the three replicates. Different letters within columns indicate significant differences according to Duncan’s Multiple Range Test (*P* ≤ 0.05)

**Fig. 3:**
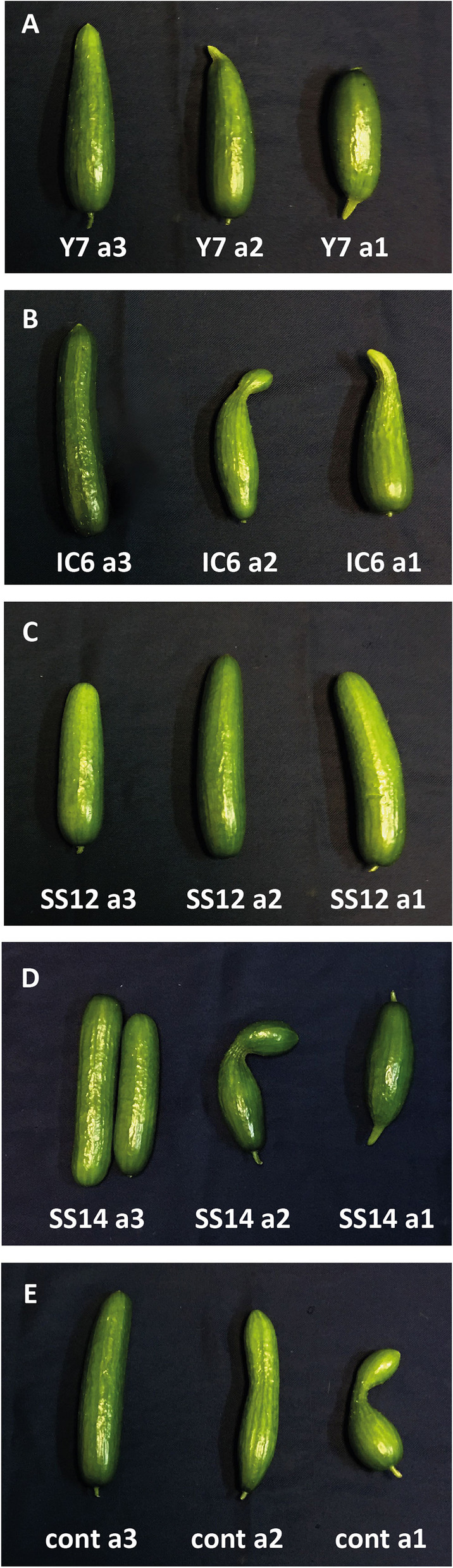
The effects of strain inoculation and different level of NPK fertilizer on morphological characters of cucumber fruits. Different levels of NPK fertilizers are a1 (50% = 1 g/L) a2 (75% = 1.5 g/L) and a3 (100% = 2 g/L) based on recommended amount of different growth levels of cucumber (vegetative growth, flowering and fruiting)

Analysis of variance for various crop growth promotion parameters showed significant differences among the treatments with different strains of *Streptomyces* inoculation, ratio fertilization, and their interactive effects. The results of comparing the means of all the characters with substantial differences in interactive effects were displayed as a bar graph with different letters in each column. As depicted in Fig. 2A, using PGPR increased the number of marketable fruits at various fertilizer levels. The strain SS12 produced the most cucumbers (about 29% increase) among all treatments containing 75% fertilizer level compared to 100% NPK fertilizer. In most treatments, fruit fresh weight (FFW) did not increase compared to the control. However, SS14 strain inoculation at a fertilizer level of 75% yielded the highest FFW (Fig. 2B). Skin thickness was increased after inoculation but there was no significant difference in different NPK levels under inoculation (Fig. 2C). After inoculation with SS12 and Y7 strains in 75% NPK fertilizer, the yield increased by 70% and 30%, respectively. Furthermore, the SS14 strain increased yield by the same amount as the SS12 strain when using 100% NPK fertilizer (without a reduction in fertilizer usage), which is not a concern of ours (Fig. 2D). Figure 2E depicts the impact of various fertilizer concentrations and PGPR strains on the firmness of cucumbers. Compared to the control, the SS12 and Y7 treatments with 75% NPK fertilizer produced the firmest fruit. All inoculated treatments increased fruit dry weight percentage (% FDW) by half the amount of NPK fertilizer compared to the control, but adding fertilizers did not increase the % FDW (Fig. 2F). Conversely, there is a sympathy between control treatments and fertilizer addition. The interaction effects of fertilization and bio-inoculation on fruit yield were found to be significant. Overall, inoculation increased yield, but SS12 produced the highest yield with 75% NPK compared to the control. Adding more fertilizer increased the diameter of the fruit, with the smallest diameter belonging to 50 and 75% of fertilizer in the SS12 treatment (Fig. 2G). The results of PGPR inoculation on root dry weight percentage (% RDW) showed that inoculation increased % RDW totally, but IC6 and SS12 inoculation recorded the highest rate of root dry weight under 75% fertilization (Fig. 2H). Figure 3 depicts fruit images from various treatments of the second test.

#### Chlorophyll a, b, total, and carotenoid content of cucumber leaves

**Fig. 4:**
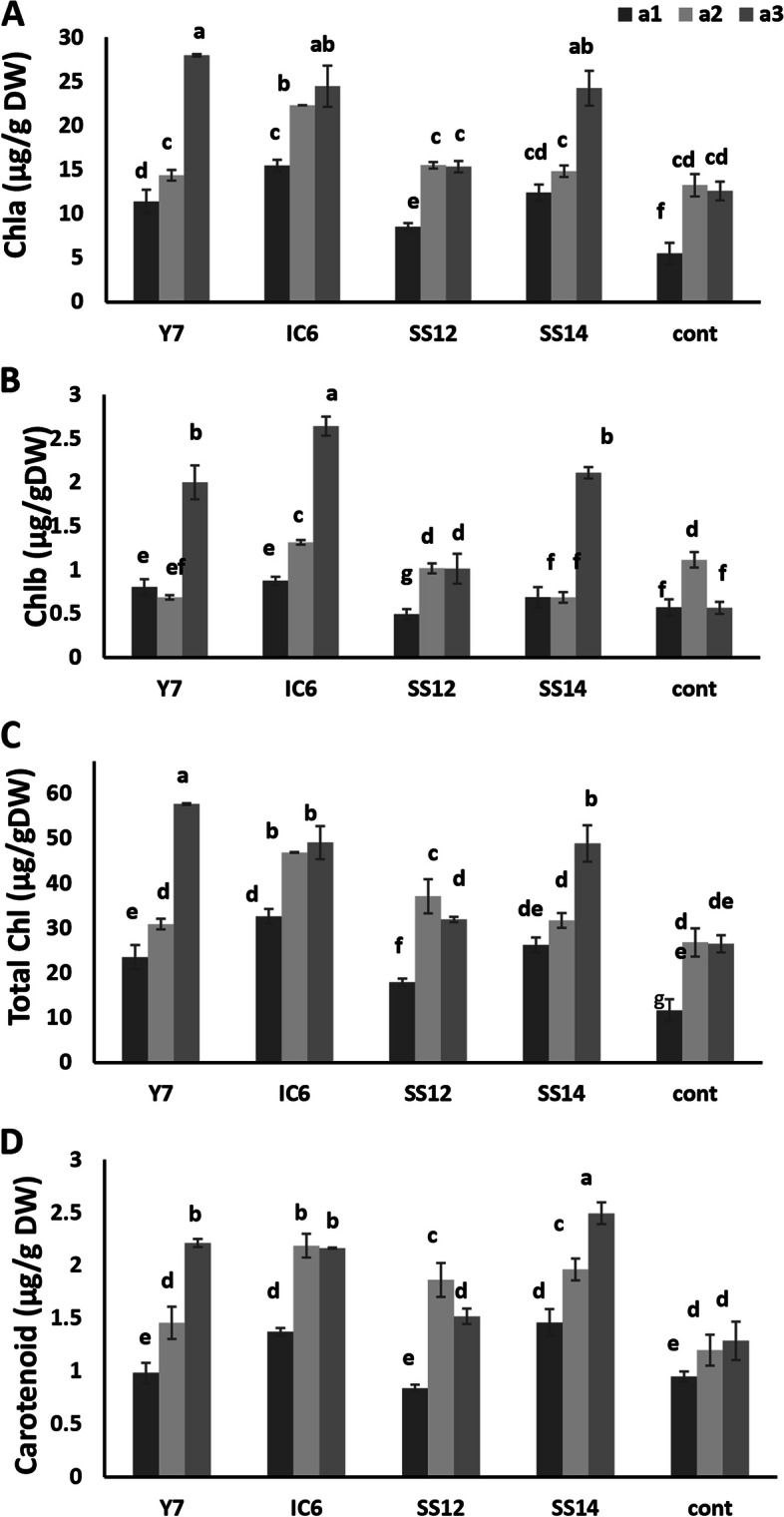
The effect of different quantities of NPK fertilizer and PGPR bacteria on chlorophyll and carotenoid of cucumber leaves in the productive phase. **A** Variation of Chla content of inoculated cucumber leaves. **B** Variation of Chlb content of inoculated cucumber leaves. **C** Variation of total Chl content of inoculated cucumber leaves. **D** Variation of carotenoid content of inoculated cucumber leaves. Different level of NPK fertilizers is a1 (50% = 1 g/L) a2 (75% = 1.5 g/L) and a3 (100% = 2 g/L) based on recommended amount of different growth level of cucumber (vegetative growth, flowering, and fruiting). Values represent means of three replicates; bars represent standard deviation of the three replicates. Different letters within columns indicate significant differences according to Duncan’s Multiple Range Test (*P* ≤ 0.05)

Detection of photosynthetic pigments showed that inoculation and fertilizer caused the highest increase in all pigment parameters (Fig. 4A, B). Additionally, the amount of total chlorophyll and carotenoid content increased in the SS12 strain at 75% NPK (Fig. 4C, D).

#### NPK and soluble carbohydrate content

**Fig. 5:**
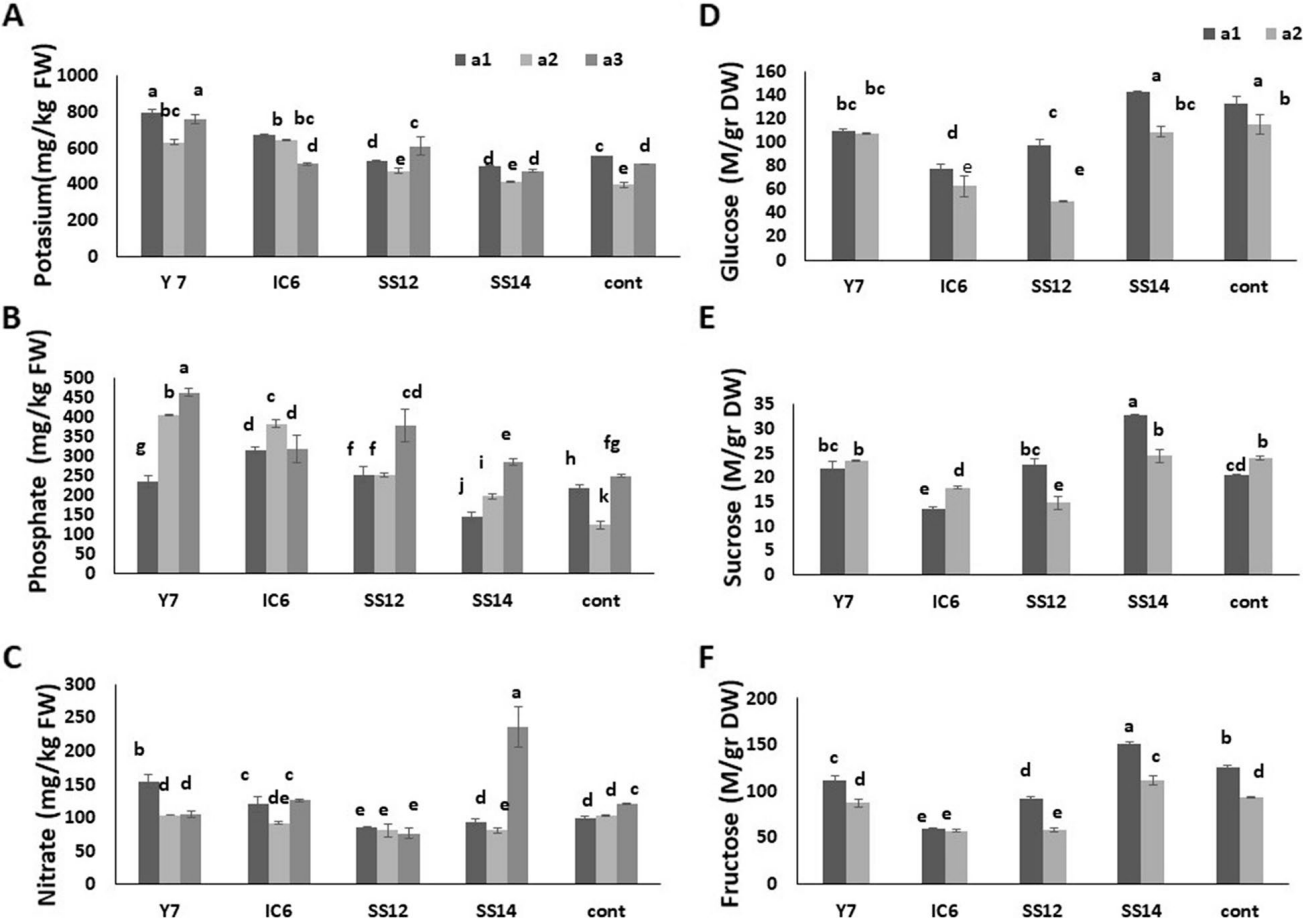
Mean comparison for effect of different NPK fertilizer quantities and PGPR bacteria on macro elements and soluble carbohydrates of fruit cucumber. **A** Variation of potassium content of inoculated cucumber fruit. **B** Variation of phosphate content of inoculated cucumber fruit. **C** Variation of nitrate content of inoculated cucumber fruit.** D** Variation of glucose content of inoculated cucumber fruit.** E** Variation of sucrose content of inoculated cucumber fruit. **F** Variation of fructose content of inoculated cucumber fruit. Different levels of NPK fertilizers are a1 (50% = 1 g/L) a2 (75% = 1.5 g/L) and a3 (100% = 2 g/L) every week based on recommended amount of different growth levels of cucumber (vegetative growth, flowering, and fruiting). In each NPK fertilizer level, a treatment co-cultivated by sand was used as a control. Values represent means of three replicates; bars represent standard deviation of the three replicates. Different letters within columns indicate significant differences according to Duncan’s Multiple Range Test (*P* ≤ 0.05)

Compared to the control, Y7 and SS12 treatments with 100% NPK fertilizer contained the greatest amount of potassium (Fig. 5A). Cucumber root inoculation with Y7, SS12, and IC6 significantly increased phosphate concentration in 75 and 100% NPK fertilizer treatments (Fig. 5B). The effect of different amounts of NPK fertilizer and PGPR bacteria on potassium, phosphate, and nitrate content of cucumber fruits revealed that SS14 and SS12 contain the highest and lowest levels of nitrate, respectively (Fig. 5C). Increasing the NPK rate increases the phosphate and potassium absorbance and nitrate content reduction of SS12 and Y7 inoculation. Overall, the most significant decrease in soluble carbohydrates (sucrose, glucose, and fructose) is 31% in cucumber fruit inoculated with SS12, and there is an increase in the abundance of fructose and sucrose in all treatments inoculated with SS14 compared to non-treated cucumber fruit (Fig. 5D, E, F).

#### Anthocyanin, carotenoid, and ascorbate content of inoculated and fertilized fruits

**Fig. 6:**
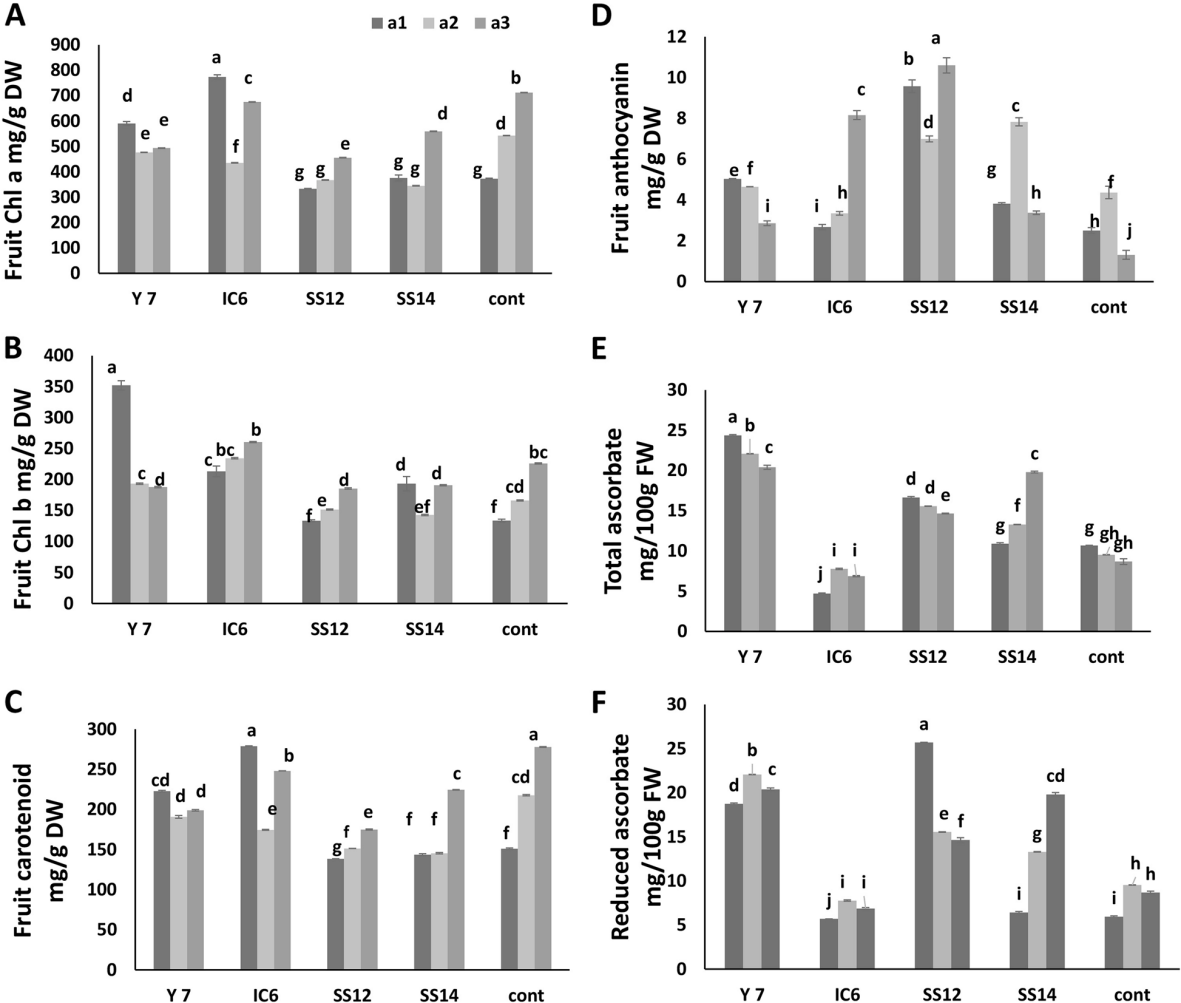
The effect of different quantities of NPK fertilizer and PGPR bacteria on some physiological characters. **A** Variation of Chla content of inoculated cucumber fruit. **B** Variation of Chlb content of inoculated cucumber fruit. **C** Variation of carotenoid content of inoculated cucumber fruit. **D** Variation of anthocyanin content of inoculated cucumber fruit. **E** and** F** Variation of total and reduced ascorbate content of inoculated cucumber fruit respectively. Values represent means of three replicates; bars represent standard deviation of the three replicates. Different levels of NPK fertilizers are a1 (50% = 1 g/L) a2 (75% = 1.5 g/L) and a3 (100% = 2 g/L) based on recommended amount of different growth levels of cucumber (vegetative growth, flowering and fruiting). Different letters within columns indicate significant differences according to Duncan’s Multiple Range Test (*P* ≤ 0.05)

The results for Chl a, Chl b, and carotenoid content in fruit for each PGPR strain treatment followed the same pattern for different NPK content. IC6 and Y7 showed an increase in Chl a, Chl b, and carotenoid content in treatments with 50% fertilizer compared to the control, but they decreased in higher NPK levels (Fig. 6A, B, C). Intriguingly, these characteristics decreased in the SS12 strain at all NPK fertilizer levels compared to the control but did not change significantly in the SS14 treatment.

All half-fertilized treatments, except for IC6, saw an increase in anthocyanin content, with the greatest variation occurring in the SS12 and SS14 treatments at 50% fertilizer (positive correlation to yield) (Fig. 6D). Intriguingly, the anthocyanin concentration in Y7 decreased as fertilizer levels increased. Total and reduced ascorbate levels rose after SS12 and Y7 inoculation (Fig. 6E, F).

#### Correlation analysis between yield and qualitative and quantitative characteristics

There was a significant positive relationship between yield and fruit number (*r* = 0.3), anthocyanin production (*r* = 0.31), skin thickness (*r* = 0.46), and firmness (Fmax) (*r* = 0.33) and a negative correlation between yield and fruit diameter (*r* = 0.57). Moreover, there was a significant positive correlation between fruit no. and height have (*r* = 0.285), but there was a negative correlation with FW (*r* = 0.285) and fruit diameter and a similar relation between FW and sucrose content. Furthermore, a positive correlation was observed between fruit diameter and total Chl. Moreover, root dry weight was significantly correlated with leaf Chl a (*r* = 0.27) and total Chl (*r* = 0.33) (Table 4). There was a significant positive correlation between leaf Chl a, Chl b and total Chl pairwise. There was a strong negative correlation between potassium, phosphorus and yield. In addition, the same relationship exists between the number of fruits and all soluble carbohydrates (Suc, Glu, Fru).

**Table 4:** Correlations matrix of characters related to (a) yield and cucumber fruits qualitative characteristics and (b) yield and carbohydrate content /chemical fertilizer components in cucumber fruits

**(a)**													
	Yeild	Fruit No	FW	Skin Th	Fruit D	Fmax	RDW	Cart fruit	Ant fruit	Chla	Chlb	Total	
Yeild	1												
Fruit No	.308^*^	1											
FW	- .89	-.285^*^	1										
Skin Th	.463^*^	.167	-.01	1									
Fruit D	-.577^*^	-.413^*^	.227	-.305	1								
Fmax	.33^*^	-.182	.06	.30^*^	-.103	1							
RDW	.076	-.012	-.102	.125	-.03	.101	1						
Cart fruit	-.025	.051	-.198	-.434	.134	.082	- .03	1					
Ants fruit	.310^*^	.127	-.003	.049	-.24	.195	.015	-.252	1				
Chla	.187	.056	.096	.12	.057	.133	.278^*^	.272^*^	.019	1			
Chlb	.065	.174	.011	-.09	-.04	-.027	.050	.330	-.026	.775^**^	1		
Total	.231	.035	.136	.415^**^	.012	.209	.330^*^	.254	.060	.961^**^	.722^**^	1	
**(b)**													
	Yeild	Fruit No	FW	Skin D	Fruit D	Fmax	RDW	NO_3_	P_3_O_4_	K	Suc	Glu	Fru
NO3	.025	.279	-.128	.392^**^	-.08	-.164	-.06	1					
P3O4	-.391^**^	-.07	-.085	.076	.159	.137	-.11	.32^*^	1				
K	-.42^**^	-.105	-.119	.056	.191	.182	-.079	.277	.628^**^	1			
Suc	.044	-.419^*^	.793^**^	.184	.075	-.224	-.165	.114	-.372	-.398	1		
Glu	-.195	-.325	.539^**^	.048	.188	-.488^**^	-.007	-.109	-.524^**^	-.400^**^	.768^**^	1	
Fru	-.016	-.244	.573^**^	.268	.109	-.445^*^	-.126	-.033	-.566^**^	-.516^**^	.802^**^	.905^**^	1

*Fruit No* Fruit number, *FW* Fresh weight, *Skin Th* Skin Thickness, *Fruit D* Fruit diameter, *Fmax* Maximal force, *RDW* Root dry weight, *Cart fruit* Carotenoid fruits, *Ants Fruit* Anthocyanin fruits, *Chla* Chlorophyll a, *Chlb* Chlorophyll b, *Suc* Sucrose content, *Glu* Glucose content, *Fru* Fructose content. The values marked with an (*P*< 0.05) or two (*P*< 0.01) asterisks are significantly  correlation coefficient

#### Enzyme activity and their gene expression

**Fig. 7:**
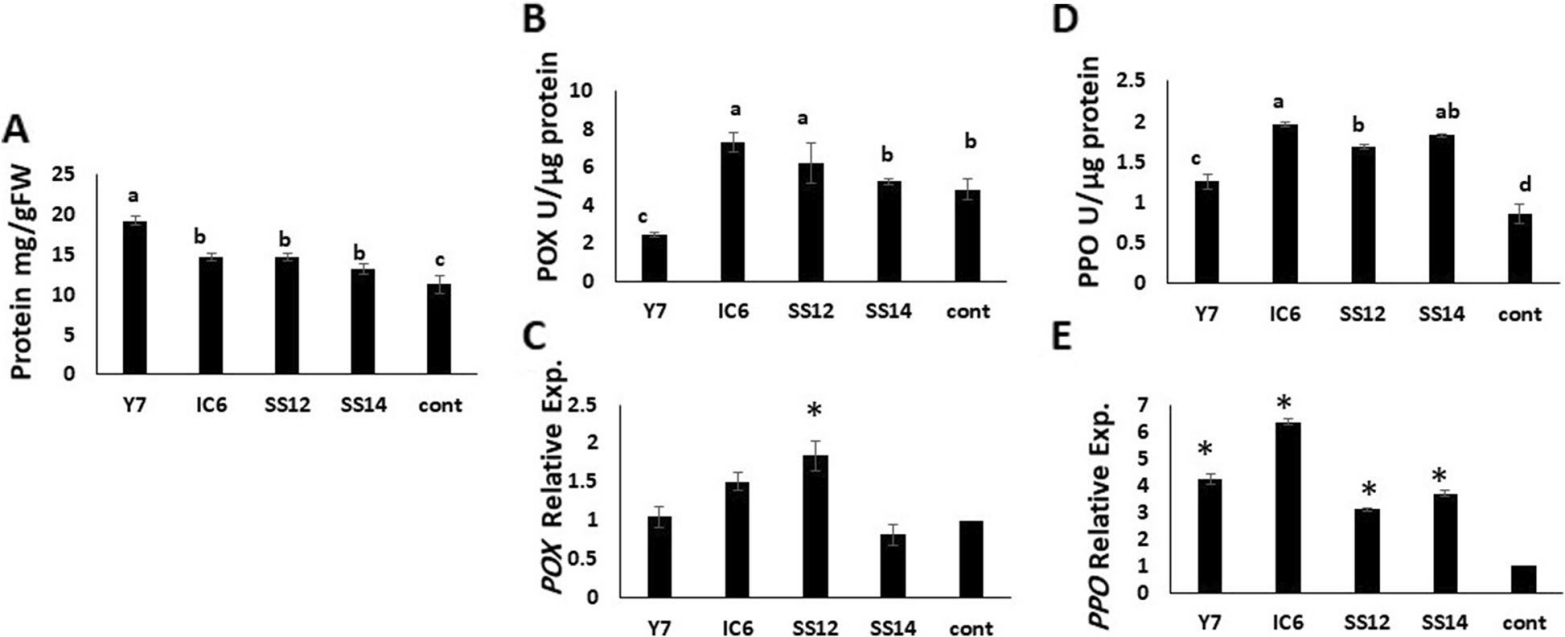
The effect of different PGPR bacteria on **A** protein content, **B**, **C** Peroxidase (POX) and Poly Phenol Oxidase (PPO) activity enzyme, and **D**, **E** their gene expression in cucumber leaf after 1 week cultivation. Values represent means of four replicates; bars represent standard deviation of the four replicates. Different letters within columns indicate significant differences according to Duncan’s Multiple Range Test (*P* ≤ 0.05)

After 1 week of inoculation, the protein content of cultivated leaf cucumbers showed a 41% and 14% increase for the Y7 and IC6, SS12 treatments, respectively (Fig. 7A). In the leaves of plants treated with strains IC6 (50%) and SS12 (29%), POX activities increased by 50%, whereas they decreased by 50% in plants inoculated with strain Y7 (Fig. 7B). All treatments increased PPO activity compared to the control (Fig. 7D). The results of gene expression analysis revealed that the level of POX increased 1.5-fold in IC6 and twofold in SS12 (Fig. 7C). Plants treated with *Streptomyces* strains had higher *PPO* gene expression levels than untreated plants (Fig. 7E). Moreover, IC6 cultivation treatment can induce *PPO* gene expression by a factor greater than six, even though all PGPR treatments upregulated PPO compared to the control.

#### Plant growth regulators and their gene expression

**Fig. 8:**
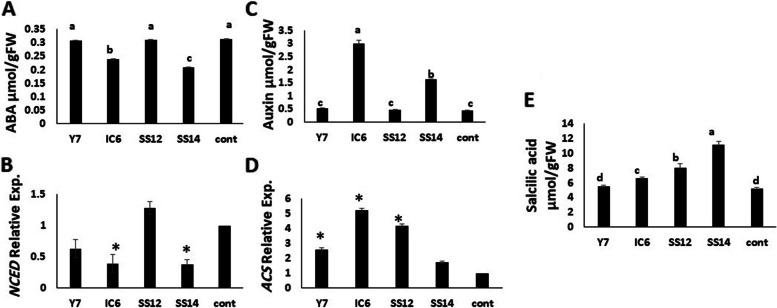
The effect of different PGPR bacteria on **A** Abscisic acid **C** IAA **E** Salicylic acid and **B** gene expression of *NCED* and **D ***ACS* gene expression in cucumber leaf after 1-week cultivation. Values represent means of four replicates; bars represent standard deviation of the three replicates. Different letters and asterisks (*) within columns indicate significant differences according to Duncan’s Multiple Range Test (*P* ≤ 0.05)

After 1 week, ABA content and gene expression of *NCED3* (one of the key genes involved in ABA biosynthesis) decreased in SS14 and IC6 leaves cultivated with PGPR in cucumber. On the contrary, both PGPR treatments increased IAA concentration. The expression of the *ACS* gene increased in every treatment except for SS14. Compared to the control, the salicylic acid content of SS14, SS12, and IC6 treatments increased by 220%, 75%, and 50%, respectively (Fig. 8).

## Discussion

This study aimed to enhance the growth, yield and quality characteristics of cucumber fruits using reduced fertilizers by using specific PGPRs from cucumber fields. In addition, several effective direct and indirect biochemical features were discussed.

### The effects of PGPRs on growth promotion

The effects of plant growth-promoting *Streptomyces* (PGPS) as biofertilizers have been partly understudied [50, 51]. Identifying and employing region-specific microbial strains is highly recommended to maximize the effectiveness of utilized PGPR strain [52]. Our results showed that cucumber_specific strains with various rates of PGP properties and antagonistic activity increased root and shoot dry weight (Tables 2 and 3). Cucumber plants are stored for about 9 months in most greenhouses and because of the fast longitudinal growth of the plant, cucumber plants are pruned several times. In such conditions, the presence of strong roots with more volume can play vital role in plant maintenance and crop production [53]. Therefore, four strains with the highest root dry weight were selected for further treatments.

### The effects of superior strains on reducing fertilizer use and improving marketing characteristics

To reduce greenhouse fertilizer rates, we investigated the effects of three different NPK levels, four PGPR_ selected strains and their interaction effects. As a result, only significant treatments with fertilizer levels of 50% and 75% inoculated with PGPRs were introduced as superior treatments.

A collection of characteristics shows the marketability of fresh fruits such as appearance, quantity and quality.

Fruit fresh weight (FFW), cucumber height, diameter and %FDW are the effective factors determining marketable fruit. The higher (FFW in SS14), unlike the other fruits, is a negative point to reduce the number of marketable cucumber numbers in comparison to the other treatments [54].

Firmness is another important aspect of cucumber quality to reduce microorganism attacks and increase the fruit’s shelf life. Our results showed a positive relationship between firmness and fertilizer level in plants treated with SS12. This result agrees with Perez-Rodriguez et al. [55] who reported an increase in tomato fruit firmness through *Pseudomonas fluorescens* and *Azospirillum brasilense* treatment. The results may be attributable to a reduction in the polygalacturonase gene, which has been linked to increased fruit firmness in tomato fruits [56].

Several factors can control quantitative characteristics such as yield under PGPS inoculation. Our results showed that the highest level of IAA production between the mentioned strains was SS12 (Table 3). Auxin promotes stronger growth of shoots and roots and tolerance to unsuitable conditions in greenhouses. Root growth plays a crucial role in enhancing the biomass of cucumber plants treated with PGPRs, leading to an increase in yield [55]. More yield (IC6 and SS12) may be due to their ability for nitrogen fixation, phosphorus solubilization and siderophore production. Several mechanisms have been demonstrated to explain the roles of PGPRs in stimulating plant growth including (1) the ability to produce or alter the concentration of plant hormones, i. e, gibberellic acid, indole acetic acid, abscisic acid, ethylene and volatiles (2) antagonism against phytopathogenic microorganisms through the production of siderophore, antibiotics and other enzymes; (3) symbiotic nitrogen fixation; and (4) mineral phosphorus solubilization and other nutrients [57–62].

### The effects of PGPRs on photosynthetic pigments, NPK and soluble carbohydrates

Another finding is increased chlorophyll and carotenoid content of leaves in PGPR-treated plants compared to controls, which leads to increased photosynthesis and final yield in PGPR-treated cucumber plants (Figs. 2 and 4). Evaluation of NPK and soluble carbohydrates is one of the cucumber’s most important qualitative characteristics. Nitrate, the most important nitrogen source, is one of the most widely used plant compounds [63]. Despite the importance of nitrogen to plant yield and growth, nitrate accumulates in different plant parts, and the conversion of nitrate to nitrite by enzymes and bacteria produces harmful metabolites [64]. Both plant growth stimulants and microbial inoculants can influence nitrate accumulation. Nitrate accumulation can be reduced by using growth-promoting bacteria and microbial fungi alone or in combination [65]. Studying growth stimulants and nitrogen fertilizers can be particularly important in selecting the strategies needed to reduce nitrate accumulation in the edible parts of the crop. Nitrate content in cucumber fruit decreased significantly (Y7 and SS12 strains) when nitrate fertilizer was increased, whereas inoculation of the SS14 strain and nitrate fertilizer produced results similar Mitova et al. [65] and increased nitrate accumulation. The difference in the effect of various PGPR strains on nitrate absorption in fruit should be considered when using them. In other words, some PGPRs increase yield and nitrogen accumulation in edible plant parts, lowering the food value of the product. Phosphorus solubilizing bacteria increased phosphorus absorption in the fruit in our study (Table 2) but the amount of phosphate was constant in the SS14 strain due to its inability. Although, potassium fertilizer (K) helps the formation and conversion of energy and sugar through the photosynthesis process [66] but excess K caused huge cucumbers to harbor more carbohydrates and higher cucumber diameter resulted in less marketability (Table 4). The primary sugars accumulated in cucumber fruits are glucose and fructose. As shown by Alipour Kafi et al. [54] the amount of soluble sugars in cucumber fruit decreases with increasing fertilizer amount. This issue has a positive relationship with the quality acceptance of cucumbers, particularly among Iranians. The decrease is especially evident in most of our studied strains. Although the reduction of fructose in fruit has been observed even in both amounts of fertilizer in the mentioned strains, a higher level of fructose content than the control (in SS14 strain) caused sweetening of the cucumber fruit, water accumulation, and firmness reduction resulting in low-quality fruits. The negative relationship between Fmax and Glu, Fru fruit content confirmed the results above (Table 4).

### The effects of PGPs on anthocyanin, carotenoid and ascorbate content of inoculated and fertilized fruits

Cucumber fruit skin significantly impacts sales and quality, is primarily determined by the content and composition of chlorophyll, anthocyanins and carotenoids [67]. Increasing the amount of these compounds in cucumber fruits, treated with all strains, showed an effective role in increasing the green color of the fruit (Table 4, and Fig. 6) and commercial marketability [67]. Cucumber (*Cucumis sativus*) is a good source of vitamin C, which is essential in human health and nutrition [68]. Vitamin C levels increased significantly in some strains (Y7 and SS12) despite the absence of fertilizer content but it decreased with other treatments (IC6). Clearly, different strains had different effects on the quality characteristics of the cucumber, and SS12 could be introduced as the most thoroughly researched strain to improve the product’s quality. Using a consortium of Plant Growth-Promoting Rhizobacteria Strains on sweet peppers yielded the same results [69].

### The effects of PGPs on enzyme activity and their gene expression

Peroxidase (POX) is an oxidative enzyme associated with disease resistance and increases in host plants after pathogen penetration. Polyphenol oxidase (PPO) consists of a group of copper-containing enzymes widely distributed among plant species. They are involved in the plant antioxidant defense systems and reduce the harmful effects of stresses by scavenging reactive oxygen species (ROS) [70]. On the other hand, increased activity and gene expression of POX during inoculation with IC6 and SS12 and decreased enzyme activity and their expression by Y7 inoculation showed different characteristics of strains in immune induction. An increase in peroxidase (POX) activity in cucumber treated with SS12 was similar to *S.rochei* strain Y28 and pointed to a strain specific-induced systemic resistance (SS-ISR) in tomatoes. However, the PGPR-induced POX biosynthesis against Fusarium wilt disease increased significantly more than inoculation without pathogen infection [29]. Significant upregulation of *POX* and *PPO* in tomato plants inoculated with TN Vel-35(*Bacillus Subtilis*) versus respective control was also observed but *Streptomyces* inoculation on cucumber seedlings to detect PGPR effects has not yet been documented [32]. Considering the direct impact of these enzymes in strengthening the immune system, it can be concluded that PGPR inoculation alone can prime seedlings and prepare them to deal with biotic stresses. In our study, all strains increased PPO transcript levels but PPO activity did not significantly change and decreased gene expression in the Y7 strain. It is well established that the protein level does not necessarily reflect their transcript levels [71, 72]. Upregulation of POX and PPO activity enzymes demonstrated that SS12 and IC6 strains use the ISR system to protect the host plant from pathogenesis and negative environmental conditions.

### The effects of PGPRs on plant growth regulators and their gene expression

PGPRs increase growth by changing the hormone content in different parts of plants. Abscisic acid is one of the most important plant hormones produced in response to abiotic stress. Multiple strains can produce abscisic acid to protect the host plant against environmental stress such as *Pseudomonas fluorescens* Rt6M10 and *Azospirillum brasilense* Sp 245 [55]. Research results on the PGPR *Variovorax paradoxus* 5C-2 strain showed that the strain could not break down ABA in the host plant. On the other hand, some PGPR strains can break down ABA, such as *Rhodococcus* sp. P1Y, which decreased the amount of internal ABA in shoot plants [73, 74]. In our study, the reduction in leaf ABA levels of cucumbers inoculated with the SS14 and IC6 strains was due to a decrease in *NCED* gene expression, while IAA production increased root and shoot growth relative to the control. Analysis of ABA synthesis gene expression revealed a positive correlation between ABA content and gene expression in the aforementioned strains. Increasing auxin production by *Burkholderia* spp. bacteria has been shown to significantly stimulate the growth of rice plants compared to control plants [75]. The evidence indicates that auxin production could be the primary growth stimulant for the effect of some PGPR strains on the host plant in this type of study (Fig. 6). Depending on the plant species and hormone concentration, ACC as a precursor for ethylene production produced by ACS activity enzyme and ethylene can both inhibit and stimulate plant growth. Increased ethylene production may be part of the plant defense system’s response to the colonization of growth-promoting bacteria (ISR), as demonstrated in the IC6 strain by more than five times *ACS* expression and the highest defense system (Fig. 8, Table 3), but there is no significant difference between the control and SS14 treatment. The stimulation of the ACC synthase in cultivated plants by auxin-producing bacteria may also account for the increase in ethylene [76]. Strain SS12, the highest IAA producer among the studied strains, could protect cucumbers from unfavorable situations by inducing the expression of *ACS* gene.

Salicylic acid (SAL) regulates both local disease resistance mechanisms and systemic acquired resistance in plants by reducing the growth of hyphae and conidia [77, 78]. In our study, the amount of SAL in cucumber leaves inoculated with SS14 increased more than twofold after 1 week, representing the SAR system in the mentioned treatment and confirming that SS14 exhibited the highest antagonistic activity against *Phytophthora Capsici* (Table 1). Some growth-promoting strains can produce several phytohormones as shown in SS12 by SAR (SAL) and ISR system (POX, PPO, ABA and ACS).These phytohormones can have antagonistic effects and play opposite roles in the plant, such as *Bacillus licheniformis* Rt4M10 and *Pseudomonas fluorescens* Rt6M10 [79]. However, other studied strains may exhibit a different ISR and SAR system level with fewer marketable characteristics. It is difficult to predict the effects of a particular strain on a plant because it is unclear how the balance between the hormones produced by PGPR is established during colonization. To effectively use these bacteria in agriculture, it is important to understand their molecular mechanism by determining the effect of strains on the amount of plant growth regulators under various conditions.

## Conclusion

In greenhouse conditions, 17 *streptomyces* strains were used to determine the most effective one for cucumber growth. Four strains increased the root, fresh shoot, and dry weight during vegetative growth. Different strain inoculation treatments, fertilizer ratios, and interactions exhibited a statistically significant difference in our second experiment. Under 75% fertilizer, plants treated with the SS12 strain produced the greatest yield. Compared to other treatments, SS12 produced the highest quality cucumbers by decreasing the amount of nitrate, and soluble sugars and increasing the amount of antioxidants and firmness. The negative correlation between potassium intake and yield demonstrated that it induced photosynthesis and decreased marketable cucumbers by producing shorter, more watery cucumbers with a higher concentration of water-soluble carbohydrates. Antioxidant enzymes showed that SS12 and IC6 strains induced a significant increase in the activity of POX, PPO, and the expression of genes related to these two enzymes in leaves after 1-week inoculation indicating that they utilized the ISR system to protect against adverse environmental factors. The mechanism of the defense system in SS14 is distinct, and it appears that the upregulation of salicylic acid could aid the SAR system in protecting the plant from pathogens. Overall, SS12 could induce plant root growth by IAA production and supply nitrogen, phosphorus and siderophore to increase yield, reduce fertilizer consumption and prepare cucumber plants to defend themselves against unfavorable environments by combining SAR and ISR systems. Moreover, it could be utilized to promote growth in the trial cucumber field.

## Data Availability

The dataset generated during and/or analyzed during the current study is available from the corresponding author upon reasonable request.
